# Smoking and outcomes following personalized antiplatelet therapy in chronic coronary syndrome patients: A substudy from the randomized PATH‐PCI trial

**DOI:** 10.1002/clc.24214

**Published:** 2024-03-12

**Authors:** Ying Pan, Ting‐Ting Wu, Chang‐Jiang Deng, Yi Yang, Xian‐Geng Hou, Tuo Yan, Shun Wang, Ying‐Ying Zheng, Xiang Xie

**Affiliations:** ^1^ Department of Cardiology First Affiliated Hospital of Xinjiang Medical University Urumqi China; ^2^ Key Laboratory of High Incidence Disease Research in Xingjiang (Xinjiang Medical University Ministry of Education) Urumqi China; ^3^ Key Laboratory of Hypertension Research of Xinjiang Medical University Urumqi Xinjiang China

**Keywords:** antiplatelet, chronic coronary syndrome, major adverse cardiovascular and cerebrovascular events, smoking

## Abstract

**Background:**

This is a sub‐analysis of the Personalized Antithrombotic Therapy for Coronary Heart Disease after PCI (PATH‐PCI) trial in China to explore the relationship between smoking and outcomes following personalized antiplatelet therapy (PAT) in chronic coronary syndrome (CCS) patients undergoing percutaneous coronary intervention (PCI).

**Methods:**

As a single‐center, prospective, randomized controlled and open‐label trial, the PATH‐PCI trial randomized CCS patients undergoing PCI into standard group or personalized group guided by a novel platelet function test (PFT), from December 2016 to February 2018. All patients were divided into smokers and nonsmokers according to their smoking status. Subsequently, we underwent a 180‐day follow‐up evaluation. The primary endpoint was the net adverse clinical events (NACE).

**Results:**

Regardless of smoking status, in the incidence of NACE, there was a reduction with PAT but that the reductions are not statistically significant. In the incidence of bleeding events, we found no statistically significant difference between two groups (smokers: 2.0% vs. 1.4%, HR = 1.455, 95% confidence interval [CI]: 0.595−3.559, *p* = .412; nonsmokers: 2.2% vs. 1.8%, HR = 1.228, 95% CI: 0.530−2.842, *p* = .632). In smokers, PAT reduced major adverse cardiac and cerebrovascular events (MACCE) by 48.7% (3.0% vs. 5.9%, HR = 0.513, 95% CI: 0.290−0.908, *p* = .022), compared with standard antiplatelet therapy (SAT). PAT also reduced the major adverse cardiovascular events (MACE) but there was no statistically difference in the reductions (*p* > .05). In nonsmokers, PAT reduced MACCE and MACE by 51.5% (3.3% vs. 6.7%, HR = 0.485, 95% CI: 0.277−0.849, *p* = .011) and 63.5% (1.8% vs. 4.9%, HR = 0.365, 95% CI: 0.178−0.752, *p* = .006), respectively. When testing p‐values for interaction, we found there was no significant interaction of smoking status with treatment effects of PAT (*p*_int‐NACE_ = .184, *p*_int‐bleeding_ = .660).

**Conclusion:**

Regardless of smoking, PAT reduced the MACE and MACCE, with no significant difference in bleeding. This suggests that PAT was an recommendable regimen to CCS patients after PCI, taking into consideration both ischemic and bleeding risk.

## Graphical abstract

PL‐12 is a new platelet function analyzer testing the platelet maximum aggregation rate. The Personalized Antithrombotic Therapy for Coronary Heart Disease after PCI trial established that personalized antiplatelet therapy (PAT) guided by PL‐12 greatly improved the prognosis of chronic coronary syndrome (CCS) patients undergoing percutaneous coronary intervention. We explored the efficacy and safety of PAT in CCS patients under different smoking status.

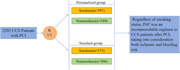

**Abbreviations**
CCS: chronic coronary syndrome
DAPT: dual antiplatelet therapy
MACCE: major adverse cardiac and cerebrovascular events
MACE: major adverse cardiovascular events
MAR: maximum aggregation rate
NACE: net adverse clinical events
PAT: personalized antiplatelet therapy
PCI: percutaneous coronary intervention
PFT: platelet function test
SAT: standard antiplatelet therapy

## 1 INTRODUCTION

Percutaneous coronary intervention (PCI) effectively improves symptoms and quality of life in patients with coronary artery disease (CAD) and may reduce cardiovascular events in a subset with higher risk CAD.^1^, ^2^ Dual antiplatelet therapy (DAPT), containing aspirin and P2Y12 receptor inhibitors, has been widely used in the treatment of patients with CAD after PCI.^3^ Recent studies have studied the use of platelet function testing (PFT) or genetic testing as a guided antiplatelet strategy.^4^, ^5^, ^6^ The results of a meta‐analysis of data from 20 743 patients showed that the guided antiplatelet strategy reduced the risk of major adverse cardiovascular events (MACE) more than standard antiplatelet therapy (SAT), including reducing the incidence of cardiac death, recurrent infarction, stroke, and in‐stent thrombosis.^7^ Smoking is a well‐recognized and preventable complex cardiovascular risk factor. In 1996, a study by Waters et al. suggested that smoking increased the risk of CAD and morbidity and mortality in CAD patients.^8^ This finding was later confirmed by a large number of studies, which further confirmed that smoking is associated with a poor prognosis of cardiovascular disease.^9^, ^10^, ^11^ Key processes involved in the initiation of atherosclerosis through smoking include endothelial dysfunction, the rise and oxidation of proatherogenic lipids, a decline in HDL (high density lipoproteins), arousal of inflammation and transition in the state of coagulation, and platelet aggregation.^12^, ^13^, ^14^, ^15^ Recently, Ramtowski et al evluated the effect of smoking and smoking cessation on platelet reactivity and aggregation with Verify Now and several other measures of platelet function. They finally found an increase in platelet reactivity and the risk of thrombotic complications in CAD patients following PCI after smoking cessation.^16^ However, no one has yet to look specifically at smokers and genetic predisposition for clopidogrel high in‐treatment platelet reactivity. We investigated this issue through sub‐analysis of the Personalized Antithrombotic Therapy for Coronary Heart Disease after PCI (PATH‐PCI) study in China, to examine the effect of smoking on the safety and efficacy of personalized antiplatelet therapy (PAT) in chronic coronary syndrome (CCS) patients after PCI.

## 2 METHODS

### 2.1 Study patients

The Personalized Antithrombotic Therapy for Coronary Heart Disease after PCI (PATH‐PCI) in CCS patients undergoing PCI (ChiCTR‐INR‐16010077) a randomized and open‐label trial, was implemented at the First Affiliated Hospital of Xinjiang Medical University. The Institutional Ethics Committee of the First Affiliated Hospital of Xinjiang Medical University had approved the protocol of this study. All patients gave written informed consent before being included in the study.

All patients aged ≥ 18 who were confirmed to have CAD were enrolled. CCS was described as a records of coronary revascularization or acute coronary syndrome (ACS)＞3 months in the past or a medical diagnosis of CAD with angiographically documented coronary artery stenosis of at least 70% diameter narrowing or a left main stenosis increased than 50% amenable to PCI as American Heart Association category. The trial's detailed methodology, inclusion and exclusion criteria, and main results were published earlier.^17^ In this exploratory sub‐analysis, we probed the effect of smoking situation on the safety and efficacy of PAT in study groups.

### 2.2 Randomization and study groups

We randomized all patients before they underwent PCI in this trial into two study groups: either (I) personalized group guided by PFT (*n* = 1146) or (II) standard group (*n* = 1139). The personalized group received PFT to evaluate the maximum aggregation rate (MAR) and took ticagrelor (90 mg twice daily) if the platelet maximum aggregation rate (MAR) > 55%, or 75 mg/day clopidogrel if the MAR ≤ 55%, as described previously.^17^ Without testing MAR, the standard group patients took the SAT for 6 months after PCI. The SAT included a clopidogrel (75‐mg/day) maintenance dosage plus an aspirin (100 mg/day) The other therapies, including angiotensin‐converting enzyme inhibitors or angiotensin II receptor blockers, b‐blockers, statins, and stents, were chosen by experienced physicians. We divided all patients into two subgroups for analysis according to their smoking status: smokers (S‐PAT, *n* = 597; S‐SAT, *n* = 573) and nonsmokers (NS‐PAT, *n* = 549; NS‐SAT, *n* = 566). For smoking, we adopted the World Health Organization (WHO) definition. Smokers were defined as those who had been smoking until admission and those who had quit within 6 months before admission (Current Smoking status). Nonsmokers included patients who stopped smoking at least 6 months earlier than inclusion in the trial.

### 2.3 PFT

Detailed detection methods have been described previously.^17^, ^18^ We used PL‐12 (SINNOWA Co., Nanjing, China) to test MAR. The MAR was calculated following formula: (1—minimum platelet count/initial platelet count) × 100%. According to the instruction manual, MAR greater than 55% was described as high‐platelet reactivity, which was highly consistent with that defined using light transmission aggregometry (LTA, *r* = 0.614, *p* < .01), Verify Now (*r* = .829, *p* < .01), and thromboelastography (TEG, *r* = .697, *p* < .01).^19^

### 2.4 Study endpoints

The primary endpoint was the net adverse clinical events (NACE), which were combination of ischemic events (cardiac death, MI, stroke, stent thrombosis, urgent revascularization) or bleeding events (Bleeding Academic Research Consortium [BARC] class ≥ 2 bleeding events, as BARC criteria defined^20^). The secondary endpoint was the individual components of the primary endpoint. All‐cause death was defined as an unequivocal, noncardiac‐cause death, as previously described.^17^ Cardiac death, all‐cause death re‐infarction, stent thrombosis and target vessel revascularization (TVR) were regarded as MACE.^14^ Major adverse cardiac and cerebrovascular events (MACCE), described as MACE, plus stroke. We followed up with patients at baseline, 1 and 6 months after recruitment through message, telephone contact or office visits.

### 2.5 Statistical analyses

We used SPSS version 22.0 (SPSS Inc.) when analyzing data. Continuous variables were performed as mean ± standard deviation (X ± S), and we compared continuous variables using *t*‐tests with normal distribution or Mann−Whitney *U*‐tests with nonnormal distribution. Categorical variables were expressed as percentages and compared by the chi‐square test or Fisher exact test. We used Kaplan–Meier survival analysis and log‐rank tests to analyze and assess the relationship between treatment and outcomes. With the adjustment of the confounders, such as sex, age, diabetes, hypertension and LDL‐C, multivariate Cox regression for interaction testing was performed to evaluate the difference of outcomes. All tests were two‐sided, and differences were statistically significant if *p* < .05.

## 3 RESULTS

### 3.1 Study population

**Figure 1:**
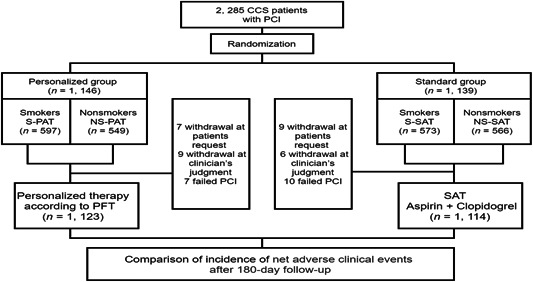
Flow chart of the PATH‐PCI study. CCS, chronic coronary syndrome; PAT, Personalized antiplatelet therapy; PCI, percutaneous coronary intervention; PFT, platelet function testing; SAT, Standard antiplatelet therapy.

We enrolled 2285 CCS patients in the PATH‐PCI study in China. We then randomly assigned 1146 patients to personalized group and 1139 patients to standard group. Patients were then subdivided into two subgroups based on smoking status (Figure 1). Table 1 showed the baseline characteristics of patients and the baseline demographics were essentially balanced. One thousand one hundrend seventy patients (51.2%) were smokers and 1904 patients (83.3%) were male. Smokers were significantly older, and more often had a diabetes and hypertension presentation. For nonsmokers, there was no difference, except in a relatively high percentage of the male study population. The angiographic of the study groups were displayed in Table S1. They were equally distributed between smokers and nonsmokers, except for stent expansion pressure, which was higher in personalized group than standard group. Stent use was more frequent in the NS‐PAT cohort, but the difference was not statistically significant due to a Type 2 error (*N* too small).

**Table 1:** Baseline characteristics of the study cohort in smokers and nonsmokers.

Characteristics	Smokers (*n* = 1, 170)				Nonsmokers (*n* = 1, 115)			
	Personalized group	Standard group	*t* or *X* ^2^	*p*	Personalized group	Standard group	*t* or *X* ^2^	*p*
	(*n* = 597)	(*n* = 573)			(*n* = 549)	(*n* = 566)		
Male, *n* (%)	466 (78.1)	467 (81.5)	2.147	.143	504 (91.8)	467 (82.5)	2.140	<.001
Age, year	60.40 ± 10.44	60.26 ± 10.26	0.232	.816	55.48 ± 10.49	56.40 ± 9.78	−1.525	.128
SBP, mmHg	132.48 ± 23.45	133.51 ± 23.53	−0.753	.452	129.03 ± 21.54	129.15 ± 21.63	−0.089	.929
HR, beats/min	76.33 ± 10.80	76.44 ± 11.86	−0.171	.864	76.42 ± 11.47	77.22 ± 10.91	−1.191	.234
Diabetes, *n* (%)	177 (29.6)	187 (32.6)	1.217	.270	134 (24.4)	148 (26.1)	0.447	.504
Hypertension, *n* (%)	355 (59.5)	354 (61.8)	0.657	.418	287 (52.3)	303 (53.5)	0.177	.674
Calcium channel blocker, *n* (%)	23 (3.9)	27 (4.7)	0.528	.467	20 (3.6)	22 (3.9)	0.046	.831
β receptor blocker, *n* (%)	131 (21.9)	116 (20.2)	0.507	.477	72 (13.1)	77 (13.6)	0.058	.810
ACEI or ARB, *n* (%)	41 (6.9)	29 (5.1)	1.696	.193	24 (4.4)	28 (4.9)	0.208	.649
IIb/IIIa inhibitors, *n* (%)	18 (3.0)	29 (5.1)	3.174	.075	15 (2.7)	23 (4.1)	1.501	.221
Statin, *n* (%)	587 (98.3)	556 (97.0)	2.164	.141	536 (97.6)	562 (99.3)	5.122	.024
BUN, mmol/L	5.52 ± 1.92	5.57 ± 1.72	−0.505	.614	5.79 ± 4.54	5.44 ± 1.55	1.734	.083
Cr, mmol/L	73.11 ± 25.91	72.26 ± 23.17	0.592	.554	77.24 ± 20.62	75.63 ± 26.38	1.130	.259
TT, s	20.73 ± 7.46	20.66 ± 2.91	0.218	.828	20.08 ± 2.66	20.43 ± 5.44	−1.360	.174
TG, mmol/L	1.74 ± 1.12	1.71 ± 1.10	0.387	.699	2.07 ± 2.30	1.95 ± 1.46	1.062	.289
TC, mmol/L	3.80 ± 1.04	3.92 ± 1.59	−1.556	.120	3.93 ± 1.09	3.91 ± 1.07	0.368	.713
HDL‐C, mmol/L	1.06 ± 0.56	1.05 ± 0.39	0.207	.836	1.03 ± 0.42	1.02 ± 0.32	0.420	.675
LDL‐C, mmol/L	2.40 ± 0.85	2.51 ± 0.98	−2.078	.038	2.51 ± 0.91	2.54 ± 0.91	−0.497	.619
apoB, mmol/L	0.78 ± 0.45	0.80 ± 0.38	−0.703	.482	0.97 ± 4.21	1.52 ± 10.60	−1.126	.260
Lp(a), mmol/L	255.84 ± 227.19	243.38 ± 212.56	0.964	.335	239.75 ± 213.52	257.06 ± 420.68	−0.860	.390
LVEF, %	63.81 ± 43.50	64.73 ± 46.35	−0.340	.734	65.14 ± 47.81	65.04 ± 45.18	0.036	.972
LVEDD, mm	49.79 ± 6.15	50.20 ± 21.84	−0.428	.669	52.28 ± 40.82	49.74 ± 4.93	1.403	.161

Abbreviations: ACEI, angiotensin‐converting enzyme inhibitor; ARB, angiotensin II receptor blocker; BUN, blood urea nitrogen; Cr, creatinine; HDL‐C, high‐density lipoprotein cholesterol; HR, heart rate; LDL‐C, low‐density lipoprotein cholesterol; Lp(a), Lipoprotein a; LVEDD, left ventricular end‐diastolic dimension; LVEF, left Ventricular Ejection Fractions; TC, total cholesterol; TG, triglyceride; TT, thrombin time; SBP, systolic blood pressure.

### 3.2 Clinical outcomes

**Figure 2:**
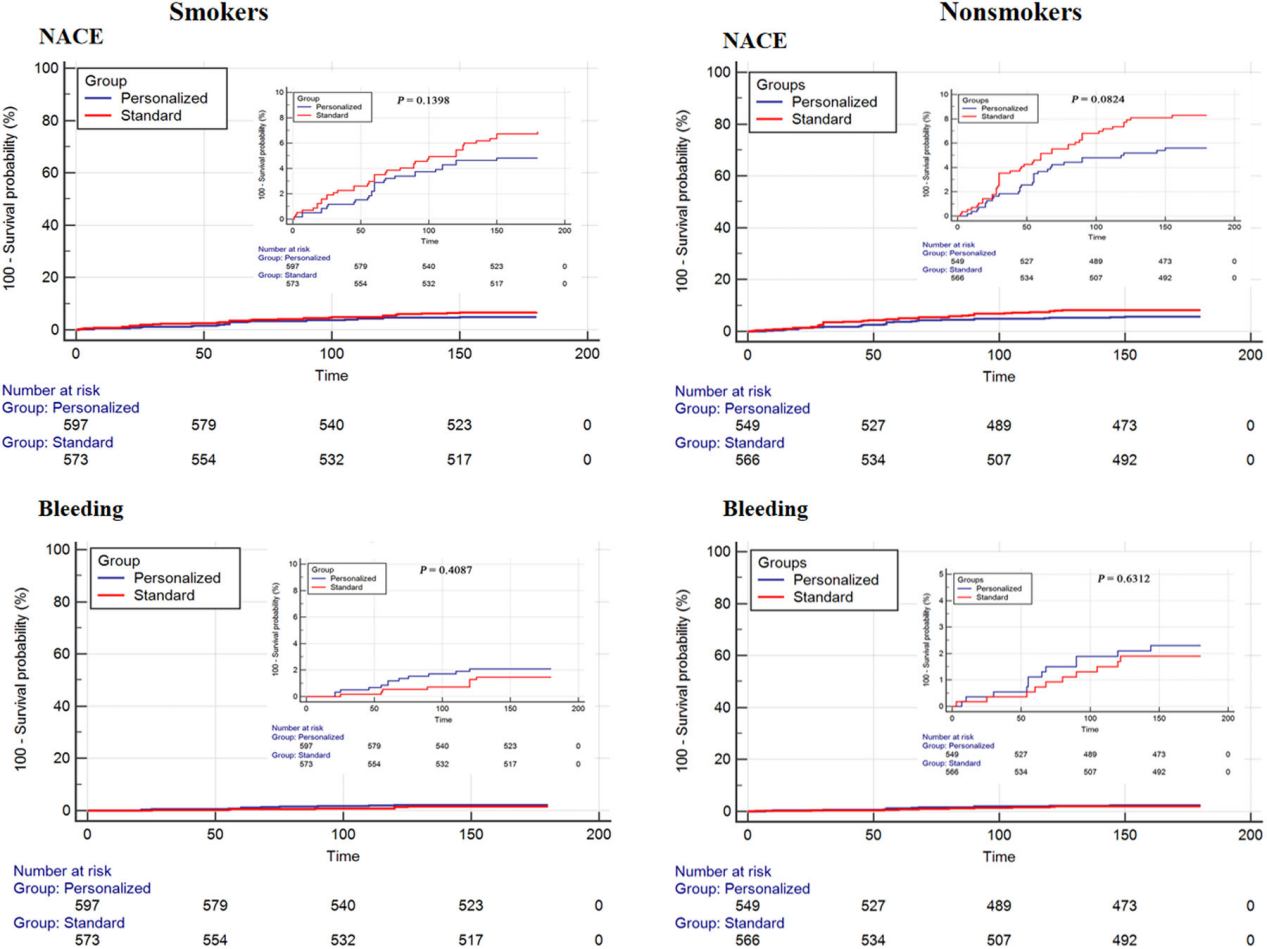
Cumulative Kaplan−Meier estimates of the time to the first adjudicated occurrence of NACE and bleeding in smokers and nonsmokers. NACE, net adverse clinical events.

Focusing on the efficacy, for the incidence of NACE, we found a reduction with PAT but that the reductions are not statistically significant (smokers, 4.7% vs. 6.8%, *p* = .142; nonsmokers, 5.5% vs. 8.1%, *p* = .085) (Figure 2 and Table 2). With regard to safety, for major bleeding, there was no difference between the groups in smokers (2.0% vs. 1.4%, *p* = .412) or nonsmokers (2.2% vs. 1.8%, *p* = .632) (Figure 2 and Table 2). Interaction tests shown that there was no significant interaction of smoking status with treatment effects of PAT (*p*_int‐NACE_ = 0.184, *p*_int‐bleeding_ = 0.660).

**Table 2:** Primary and secondary endpoints of following up for 180 days.

Endpoints	Smoking status	Personalized group	Standard group	Hazard ratio (95% CI)	*p* Value	*p* Value for interaction
NACE, *n* (%)	No	30 (5.5)	46 (8.1)	0.667 (0.421−1.057)	.085	.184
	Yes	28 (4.7)	39 (6.8)	0.695 (0.428−1.130)	.142	
MACCE, *n* (%)	No	18 (3.3)	38 (6.7)	0.485 (0.277−0.849)	.011	.051
	Yes	18 (3.0)	34 (5.9)	0.513 (0.290−0.908)	.022	
MACE, *n* (%)	No	10 (1.8)	28 (4.9)	0.365 (0.178−0.752)	.006	.287
	Yes	16 (2.7)	25 (4.4)	0.618 (0.330−1.158)	.133	
All‐cause death, *n* (%)	No	4 (0.7)	6 (1.1)	0.685 (0.193−2.428)	.558	.054
	Yes	13 (2.2)	12 (2.1)	1.051 (0.480−2.304)	.901	
Cardiac death, *n* (%)	No	2 (0.4)	4 (0.7)	0.515 (0.094−2.810)	.443	.185
	Yes	5 (0.8)	8 (1.4)	0.603 (0.197−1.844)	.375	
ST, *n* (%)	No	0 (0.0)	7 (1.2)	0.016 (0.000−6.858)	.181	.274
	Yes	5 (0.8)	8 (1.4)	0.605 (0.198−1.848)	.377	
MI, *n* (%)	No	10 (1.8)	20 (3.5)	0.511 (0.239−1.092)	.083	.384
	Yes	10 (1.7)	12 (2.1)	0.804 (0.347−1.861)	.611	
Stroke, *n* (%)	No	8 (1.5)	10 (1.8)	0.819 (0.323−2.074)	.673	.060
	Yes	2 (0.3)	9 (1.6)	0.217 (0.047−1.004)	.051	
TVR, *n* (%)	No	1 (0.2)	8 (1.4)	0.128 (0.016−1.020)	.052	.397
	Yes	3 (0.5)	7 (1.2)	0.417 (0.108−1.612)	.205	
Bleeding, *n* (%)	No	12 (2.2)	10 (1.8)	1.228 (0.530−2.842)	.632	.660
	Yes	12(2.0)	8 (1.4)	1.455 (0.595−3.559)	.412	

Abbreviations: MACCE, major adverse cardiovascular and cerebrovascular events: cardiac death, MI, ST, stroke, or urgent revascularization; MACE, major adverse cardiovascular events: cardiac death, MI, ST, or urgent revascularization; MI, myocardial infarction; NACE, net adverse clinical events; ST, stent thrombosis; TVR, target vessel revascularization.

### 3.3 Smokers

As shown in Figure S1 and Table 2, among smokers, the rates of MACCE were significantly different between S‐SAT and S‐PAT (3.0% vs. 5.9%, HR = 0.513, 95% CI, 0.290−0.908, *p* = .022). The difference in the incidence of other secondary endpoints between the groups was not statistically significant (Figure S1, S2 and Table 2; all *p* > .05). It was worth noting that the incidence of stroke was decreased in personalized group compared to standard group (0.3% vs. 1.6%, HR = 0.217, 95% CI, 0.047−1.004, *p* = .051).

### 3.4 Nonsmokers

As shown in Figure S1 and Table 2, among nonsmokers, for MACCE, the personalized group was lower than standard group (3.3% vs. 6.7%, HR = 0.485, 95% CI, 0.277−0.849, *p* = .011). In addition, we found a lower incidence of MACE in personalized group, compared with standard group (1.8% vs. 4.9%, HR = 0.365, 95% CI, 0.178−0.752, *p* = .006). However, we did not find significant difference for other secondary endpoints (Figure S1, S2 and Table 2; all *p* > .05). Notably, it showed a trend for a lower rate of TVR in personalized group (0.2% vs. 1.4%, HR = 0.128, 95% CI, 0.016−1.020, *p* = .052). These results were similar to the previously published in the primary analysis of this clinical trial.^17^ And the effectiveness of PAT was furthermore emphasized via our analyses. Cox proportional hazards model results suggested PAT was a protective factor for MACCE in CCS patients after PCI, regardless of whether they smoked (Table 3).

**Table 3:** Multivariable Cox regression analysis of MACCE according to smoking status.

Variables	Smoking status	*B*	SE	Wald	*p*	Hazard ratio (95% CI)
PAT	No	−0.715	0.291	6.063	.014	0.489 (0.277−0.864)
	Yes	−0.620	0.294	4.438	.035	0.538 (0.302−0.958)
Sex	No	−0.198	0.409	0.235	.628	0.820 (0.368−1.828)
	Yes	0.102	0.342	0.089	.766	1.107 (0.567−2.164)
Age	No	−0.012	0.014	0.701	.402	0.989 (0.962−1.016)
	Yes	−0.037	0.013	8.099	.004	0.964 (0.939−0.988)
Diabetes	No	1.540	0.280	30.220	<.001	4.667 (2.695−8.082)
	Yes	1.008	0.291	11.958	.001	2.739 (1.547−4.849)
Hypertension	No	0.061	0.283	0.047	.828	1.063 (0.611−1.851)
	Yes	−0.601	0.294	4.179	.041	0.548 (0.308−0.976)
LDL‐C	No	0.115	0.138	0.694	.405	1.121 (0.856−1.469)
	Yes	0.130	0.139	0.878	.349	1.139 (0.868−1.494)

Abbreviations: LDL‐C, low‐density lipoprotein cholesterol; PAT, personalized antiplatelet therapy.

## 4 DISCUSSION

In this study, we set out to estimate the effect of smoking on the efficacy and safety of PAT in Chinese CCS patients after PCI. While there was a significant reduction in secondary endpoints of MACE and MACCE, and no significant difference in bleeding, the primary endpoint of NACE was not significant meaning that the nonsignificant increase in bleeding offset the significant reduction in MACE. Additionally, PAT may be an independent protective factor against postoperative MACCE. PAT was an recommendable regimen to CCS patients after PCI, taking into consideration both ischemic and bleeding risk.

Smoking has been identified as a significant contributing factor to cardiovascular morbidity and mortality. It affects vascular function and hemodynamics and is linked to platelet activation.^21^ Compared with nonsmokers, smokers seemed to have a higher short‐term mortality rate after PCI.^22^ Based on the evidence of an observation study, the incidence of MACCE in patients after PCI was increased 1.060 times by smoking.^23^ Smoking increased the production of clopidogrel active metabolites by inducing CYP1A2 activity.^24^ This effect was more notable in high‐risk patients, especially those with ACS, a circumstance defined as the “smoking paradox.”^25^ The PARADOX study suggested that nonsmokers who received clopidogrel had a weaker antiplatelet effect than smokers, nonsmokers saw little or no benefit from clopidogrel treatment, especially in ACS patients.^26^ Sia et al. suggested the seemingly benefits of smoking disappeared upon adjustment.^27^ The GRAVITAS trial proposed that the more effective antiplatelet therapy may eliminate this paradox.^28^, ^29^ However, East Asian Paradox proposed that more potent P2Y12 receptor inhibitors may significantly increase the risk of severe bleeding, but do not reduce the incidence of ischemic events after PCI, as thrombogenicity may be related to race.^30^, ^31^ Recent evidence suggests the opportunity for a personalized long‐term antithrombotic monotherapy with aspirin or P2Y12 receptor inhibitor according to the patient's characteristics.^32^ These findings emphasized that clinicians should carefully consider the benefits and risks of antiplatelet drugs in different smoking situation.

Ticagrelor or prasugrel can be used when clopidogrel is ineffective or increases the risk of ischemia. And the use of ticagrelor is gradually increasing.^33^ In the multicenter, randomized, double‐blind study, named ONSET/OFFSET, compared with clopidogrel, ticagrelor was identified as a faster and more powerful P2Y12 receptor inhibitor for patients with CCS.^34^ The PLATO trial proposed that ticagrelor reduced the ischemic events without increasing major bleeding compared with clopidogrel in patients with ACS, regardless of smoking status.^35^ Later studies were again confirmed similar results in CCS patients.^36^ In the current post hoc analysis, if MAR ≥ 55%, patients received ticagrelor 90 mg twice daily plus aspirin 100 mg once daily. Regardless of smoking status, we found there was a reduction in the incidence of NACE for patients with PAT, but that the reductions are not statistically significant. And we found no statistically significant difference between two groups in the incidence of bleeding events. Among smokers, the rate of MACCE were significantly lower in PAT group than SAT group without a significant increase in bleeding events. In addition, among nonsmokers, the occurrences of MACCE and MACE were reduced with no increase in bleeding in PAT group. However, a post‐hoc analysis from the TROPICAL‐ACS trial suggested that DAPT step‐down therapy was equally safe and effective regardless of whether the patient smoked or not.^37^ When testing p‐values for interaction, we also did not find a significant interaction of smoking status with treatment effect of PAT and SAT. Interestingly, our cox proportional hazards model results suggested PAT was a protective factor for MACCE in CCS patients after PCI. Therefore, this post hoc analysis identified that although smoking influenced the vascular function and hemodynamics, it could not translate into a significant effect on the worse outcomes. These findings underscore the message about the effectiveness and safety of PAT, and revealed the harmfulness of smoking to the public and the need to quit smoking. Based on the real world, our study provided essential insights into the efficacy and safety of PAT guided by PFT in CCS patients after PCI.

## 5 LIMITATIONS

Some potential limitations need to be considered. First, the PATH‐PCI study is an open‐label, single‐center study in a single ethnic group that was predominantly male, the results may not be (and probably are not) generalizable to the other populations. It is necessary to conduct multicenter and large‐sample clinical trials to further investigate the efficacy and safety of personalized therapy based on platelet function. Second, we performed a subgroup analysis according to smoking status, which is a post hoc analysis with all its inherent limitations. Third, the smoking status in this study was self‐reported by patients, and there was no biochemical test involving cigarette components. Finally, we measured platelet function in patients with the PL‐12 platelet function analyzer only in the initial state, did not retest during subsequent follow‐ups, and did not study the pharmacokinetics of clopidogrel or ticagrelor.

## 6 CONCLUSION

To sum up, smoking is a modifiable risk factor. Regardless of smoking, PAT reduced the MACE and MACCE, with no significant difference in bleeding. This suggests that PAT was an recommendable regimen to CCS patients after PCI, taking into consideration both ischemic and bleeding risk.

## AUTHOR CONTRIBUTIONS

Ying Pan and Ting‐Ting Wu drafted the manuscript, designed the study and provided methodological expertize. Tuo Yan, Chang‐Jiang Deng, Yi Yang, Shun Wang, and Xian‐Geng Hou drafted the tables and figures and performed the statistical analysis. Xiang Xie and Ying‐Ying Zheng designed the study and made critical revision of the manuscript. All authors contributed to the acquisition of data and final approval of the version to be published. All authors agreed to be accountable for all aspects of the work.

## CONFLICT OF INTEREST STATEMENT

The authors declare no conflict of interest.

## Supporting information


**Figure S1**. Cumulative Kaplan‐Meier estimates of the time to the first adjudicated occurrence of MACCE and MACE in smokers and nonsmokers. (Note: MACCE, major adverse cardiovascular and cerebrovascular events; MACE, major adverse cardiovascular events).

**Figure S2**. Cumulative Kaplan‐Meier estimates of the time to the first adjudicated occurrence of all‐cause death and cardiac death in smokers and nonsmokers.

Supporting information.

## Data Availability

Data of this study were available from the corresponding author upon request.

## References

[1] Lawton JS , Tamis‐Holland JE , Bangalore S , et al. 2021 ACC/AHA/SCAI guideline for coronary artery revascularization. JACC. 2022;79(2):e21‐e129. 34895950 10.1016/j.jacc.2021.09.006

[2] Cook CM , Ahmad Y , Howard JP , et al. Impact of percutaneous revascularization on exercise hemodynamics in patients with stable coronary disease. JACC. 2018;72(9):970‐983. 30139442 10.1016/j.jacc.2018.06.033 PMC6580361

[3] Task Force Members , Montalescot G , Sechtem U , et al. 2013 ESC guidelines on the management of stable coronary artery disease: the task force on the management of stable coronary artery disease of the European Society of Cardiology[J]. Eur Heart J. 2013;34(38):2949‐3003. 23996286 10.1093/eurheartj/eht296

[4] Sibbing D , Aradi D , Alexopoulos D , et al. Updated expert consensus statement on platelet function and genetic testing for guiding P2Y12 receptor inhibitor treatment in percutaneous Coronary Intervention. JACC. 2019;12(16):1521‐1537. 31202949 10.1016/j.jcin.2019.03.034

[5] Moon JY , Franchi F , Rollini F , et al. Role of genetic testing in patients undergoing percutaneous coronary intervention. Exp Rev Clin Pharmacol. 2018;11(2):151‐164. 10.1080/17512433.2017.1353909 PMC5771818 28689434

[6] Franchi F , Rollini F , Cho JR , et al. Platelet function testing in contemporary clinical and interventional practice[J]. Curr Treat Options Cardiovasc Med. 2014;16(5):1‐22. 10.1007/s11936-014-0300-y 24652579

[7] Galli M , Benenati S , Capodanno D , et al. Guided versus standard antiplatelet therapy in patients undergoing percutaneous coronary intervention: a systematic review and meta‐analysis. Lancet. 2021;397(10283):1470‐1483. 33865495 10.1016/S0140-6736(21)00533-X

[8] Waters D , Lespe' rance J , Gladstone P , et al. Effects of cigarette smoking on the angiographic evolution of coronary atherosclerosis: A Canadian coronary atherosclerosis intervention trial (CCAIT) substudy. Circulation. 1996;94(4):614‐621. 8772679 10.1161/01.cir.94.4.614

[9] Ding N , Sang Y , Chen J , et al. Cigarette smoking, smoking cessation, and Long‐Term risk of 3 Major Atherosclerotic diseases. JACC. 2019;74(4):498‐507. 31345423 10.1016/j.jacc.2019.05.049 PMC6662625

[10] Rigotti NA , McDermott MM . Smoking cessation and cardiovascular disease. JACC. 2019;74(4):508‐511. 31345424 10.1016/j.jacc.2019.06.003

[11] Mayne SL , Widome R , Carroll AJ , et al. Longitudinal associations of Smoke‐Free policies and incident cardiovascular disease: CARDIA study. Circulation. 2018;138(6):557‐566. 29735485 10.1161/CIRCULATIONAHA.117.032302 PMC6202173

[12] Kang J , Kim HL , Seo JB , Lee JY , Moon MK , Chung WY . Endothelial function estimated by digital reactive hyperemia in patients with atherosclerotic risk factors or coronary artery disease. Heart Vessels. 2018;33(7):706‐712. 29352760 10.1007/s00380-018-1118-4

[13] Messner B , Bernhard D . Smoking and cardiovascular disease: mechanisms of endothelial dysfunction and early atherogenesis. Arterioscler Thromb Vasc Biol. 2014;34(3):509‐515. 24554606 10.1161/ATVBAHA.113.300156

[14] Aksoy S , Cam N , Gurkan U , et al. Oxidative stress and severity of coronary artery disease in young smokers with acute myocardial infarction. Cardiol J. 2012;19(4):381‐386. 22825899 10.5603/cj.2012.0069

[15] Laytragoon Lewin N , Karlsson JE , Robinsson D , et al. Influence of single nucleotide polymorphisms among cigarette smoking and non‐smoking patients with coronary artery disease, urinary bladder cancer and lung cancer[J]. PLoS One. 2021;16(1):e0243084. 33507988 10.1371/journal.pone.0243084 PMC7842923

[16] Ramotowski B , Undas A , Budaj A . Altered platelet reactivity, coagulation, endothelial and inflammatory markers early after smoking cessation verified with cotinine plasma concentration[J]. J Thromb Thrombolysis. 2023;56(1):75‐81. 37138182 10.1007/s11239-023-02819-5 PMC10284957

[17] Zheng YY , Wu TT , Yang Y , et al. Personalized antiplatelet therapy guided by a novel detection of platelet aggregation function in stable coronary artery disease patients undergoing percutaneous coronary intervention: a randomized controlled clinical trial. Euro Heart J Cardiovas Pharmacother. 2020;6(4):211‐221. 10.1093/ehjcvp/pvz059 31603191

[18] Pan Y , Wu TT , Hou XG , et al. Age and outcomes following personalized antiplatelet therapy in chronic coronary syndrome patients: a post hoc analysis of the randomized PATH‐PCI trial. Platelets. 2023;34(1):2206915. 37154019 10.1080/09537104.2023.2206915

[19] Guan J , Cong Y , Ren J , et al. Comparison between a new platelet count drop method PL‐11, light transmission aggregometry, VerifyNow aspirin system and thromboelastography for monitoring short‐term aspirin effects in healthy individuals. Platelets. 2015;26(1):25‐30. 24433273 10.3109/09537104.2013.865835

[20] Amin AM , Sheau Chin L , Teh CH , et al. Pharmacometabolomics analysis of plasma to phenotype clopidogrel high on treatment platelets reactivity in coronary artery disease patients. Eur J Pharm Sci. 2018;117:351‐361. 29526765 10.1016/j.ejps.2018.03.011

[21] Erhardt L . Cigarette smoking: an undertreated risk factor for cardiovascular disease. Atherosclerosis. 2009;205(1):23‐32. 19217623 10.1016/j.atherosclerosis.2009.01.007

[22] Parasuraman S , Zaman AG , Egred M , et al. Smoking status and mortality outcomes following percutaneous coronary intervention. Euro J Preven Cardiol. 2021;28(11):1222‐1228. 10.1177/2047487320902325 33611373

[23] Zhang J , Hao JY , Jing R , et al. Current trends in optimal medical therapy after PCI and its influence on clinical outcomes in China. BMC Cardiovasc Disord. 2021;21(1):258. 34039268 10.1186/s12872-021-02052-z PMC8157424

[24] Kroon LA . Drug interactions with smoking. Am J Health Syst Pharm. 2007;64(18):1917‐1921. 17823102 10.2146/ajhp060414

[25] Gagne JJ , Bykov K , Choudhry NK , et al. Effect of smoking on comparative efficacy of antiplatelet agents: systematic review, meta‐analysis, and indirect comparison[J]. BMJ. 2013;347. 10.1136/bmj.f5307 PMC3775704 24046285

[26] Gurbel PA , Bliden KP , Logan DK , et al. The influence of smoking status on the pharmacokinetics and pharmacodynamics of clopidogrel and prasugrel. JACC. 2013;62(6):505‐512. 23602770 10.1016/j.jacc.2013.03.037

[27] Sia CH , Ko J , Zheng H , et al. Association between smoking status and outcomes in myocardial infarction patients undergoing percutaneous coronary intervention. Sci Rep. 2021;11(1):6466. 33742073 10.1038/s41598-021-86003-w PMC7979717

[28] Price MJ . Standard‐ vs High‐Dose clopidogrel based on platelet function testing after percutaneous coronary intervention: the GRAVITAS randomized trial. JAMA. 2011;305(11):1097‐1105. 21406646 10.1001/jama.2011.290

[29] Reed GW , Cannon CP , Waalen J , et al. Influence of smoking on the antiplatelet effect of clopidogrel differs according to clopidogrel dose: insights from the GRAVITAS trial. Catheter Cardiovasc Interv. 2017;89(2):190‐198. 26909669 10.1002/ccd.26428

[30] Jeong YH . “East asian paradox”: challenge for the current antiplatelet strategy of “one‐guideline‐fits‐all races” in acute coronary syndrome[J]. Curr Cardiol Rep. 2014;16(5):1‐8. 10.1007/s11886-014-0485-4 24668607

[31] Kim HK , Tantry US , Smith Jr. SC , et al. The east asian paradox: an updated position statement on the challenges to the current antithrombotic strategy in patients with cardiovascular disease. Thromb Haemost. 2021;121(04):422‐432. 33171520 10.1055/s-0040-1718729

[32] Gragnano F , Cao D , Pirondini L , et al. P2Y12 inhibitor or aspirin monotherapy for secondary prevention of coronary events. JACC. 2023;82(2):89‐105. 37407118 10.1016/j.jacc.2023.04.051

[33] Piqueras‐Flores J , Jurado‐Román A , López‐Lluva MT , Sánchez‐Pérez I , Abellán‐Huerta J , Lozano‐Ruiz Poveda F . Efficacy and safety of loading doses with P2Y12‐Receptor antagonists in patients without dual antiplatelet therapy undergoing elective coronary intervention. J Cardiovasc Pharmacol. 2019;73(1):56‐59. 30383607 10.1097/FJC.0000000000000632

[34] Gurbel PA , Bliden KP , Butler K , et al. Randomized Double‐Blind assessment of the ONSET and OFFSET of the antiplatelet effects of ticagrelor versus clopidogrel in patients with stable coronary artery disease: the ONSET/OFFSET study. Circulation. 2009;120(25):2577‐2585. 19923168 10.1161/CIRCULATIONAHA.109.912550

[35] Cornel JH , Becker RC , Goodman SG , et al. Prior smoking status, clinical outcomes, and the comparison of ticagrelor with clopidogrel in acute coronary syndromes—insights from the PLATelet inhibition and patient outcomes (PLATO) trial. Am Heart J. 2012;164(3):334‐342. 22980299 10.1016/j.ahj.2012.06.005

[36] Li J , Qiu H , Yan L , et al. Efficacy and safety of ticagrelor and clopidogrel in patients with stable coronary artery disease undergoing percutaneous coronary intervention. J Atheroscler Thromb. 2021;28(8):873‐882. 32908113 10.5551/jat.57265 PMC8326171

[37] Orban M , Trenk D , Geisler T , et al. Smoking and outcomes following guided de‐escalation of antiplatelet treatment in acute coronary syndrome patients: a substudy from the randomized TROPICAL‐ACS trial. Euro Heart J Cardiovasc Pharmacothe. 2020;6(6):372‐381. 10.1093/ehjcvp/pvz084 31855244

