# Supplementary figures and images for: Smoking and outcomes following personalized antiplatelet therapy in chronic coronary syndrome patients: A substudy from the randomized PATH‐PCI trial
Source: Clin Cardiol. 2024 Mar 12;47(3):e24214. doi: 10.1002/clc.24214 (PMC10933083; doi:10.1002/clc.24214)

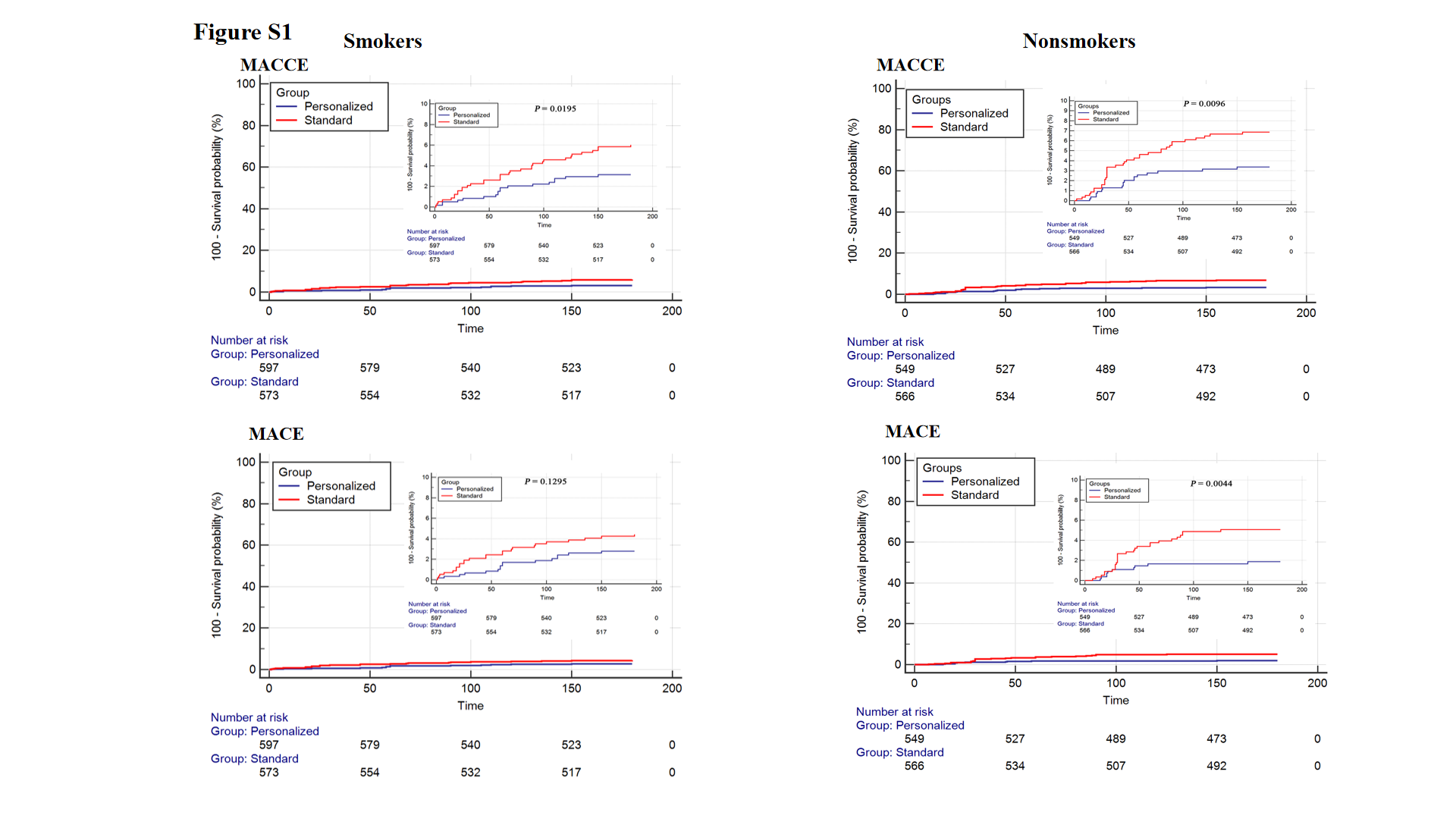

Supplement: Supplementary file 1 — Figure S1. Cumulative Kaplan‐Meier estimates of the time to the first adjudicated occurrence of MACCE and MACE in smokers and nonsmokers. (Note: MACCE, major adverse cardiovascular and cerebrovascular events; MACE, major adverse cardiovascular events). [file CLC-47-e24214-s001.tif]

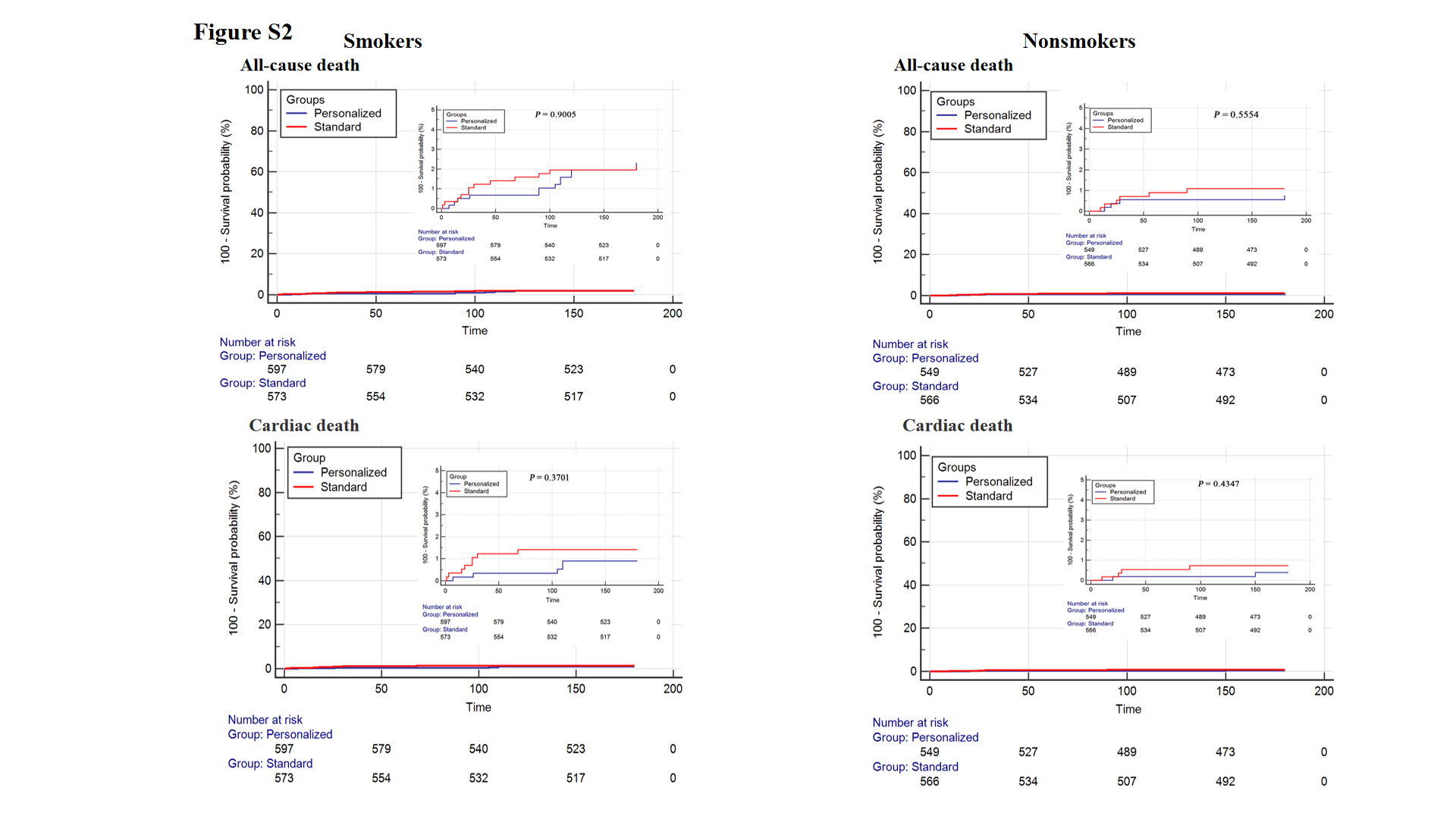

Supplement: Supplementary file 2 — Figure S2. Cumulative Kaplan‐Meier estimates of the time to the first adjudicated occurrence of all‐cause death and cardiac death in smokers and nonsmokers. [file CLC-47-e24214-s003.tif]
