# Supplementary material for: Smoking and outcomes following personalized antiplatelet therapy in chronic coronary syndrome patients: A substudy from the randomized PATH‐PCI trial
Source: Clin Cardiol. 2024 Mar 12;47(3):e24214. doi: 10.1002/clc.24214 (PMC10933083; doi:10.1002/clc.24214)
Supplement: Supplementary file 3 — Supporting information. [file CLC-47-e24214-s002.docx]

| **Table S1. Angiographic and procedural characteristics of the study cohort in smokers and nonsmokers** | | | | | | | | |  |
| --- | --- | --- | --- | --- | --- | --- | --- | --- | --- |
| Characteristics | Smokers (*n* = 1, 170) | | | | Nonsmokers (*n* = 1, 115) | | | |  |
|  | Personalized group | Standard group | *t* or *X^2^* | P | Personalized group | Standard group | *t* or *X^2^* | P |  |
|  | (*n* = 597) | (*n* = 573) |  |  | (*n* = 549) | (*n* = 566) |  |  |  |
| Number of target lesions | 1.60 ± 0.80 | 1.58 ± 0.81 | 0.395 | 0.693 | 1.64 ± 0.79 | 1.56 ± 0.79 | 1.559 | 0.119 |  |
| LM, *n* (%) | 21(3.5) | 25(4.4) | 0.553 | 0.457 | 24(4.4) | 22(3.9) | 0.166 | 0.684 |  |
| CTO, *n* (%) | 23(3.9) | 15(2.6) | 1.419 | 0.234 | 18(3.3) | 18(3.2) | 0.009 | 0.926 |  |
| Stent expansion pressure, atm | 11.89 ± 3.03 | 12.80 ± 3.42 | - 4.802 | < 0.001 | 12.04 ± 3.01 | 13.05 ± 3.76 | - 4.928 | < 0.001 |  |
| Post-dilation, *n* (%) | 554(92.8) | 523(91.3) | 0.927 | 0.336 | 510(92.9) | 524(92.6) | 0.041 | 0.839 |  |
| Diameter of stents, mm | 2.83 ± 0.39 | 2.83 ± 0.38 | - 0.101 | 0.920 | 2.86 ± 0.40 | 2.83 ± 0.37 | 1.214 | 0.225 |  |
| Length of stents, mm | 26.58 ± 6.81 | 26.49 ± 7.37 | 0.215 | 0.830 | 26.23 ± 7.05 | 26.31 ± 7.18 | - 0.200 | 0.842 |  |
| Number of stents | 1.17 ± 0.46 | 1.14 ± 0.45 | 1.049 | 0.295 | 1.21 ± 0.53 | 1.12 ± 0.43 | 3.039 | 0.002 |  |
| Procedure time, min | 32.87 ± 11.40 | 31.92 ± 9.42 | 1.544 | 0.123 | 33.29 ± 11.83 | 33.05 ± 9.81 | 0.378 | 0.706 |  |
| Abbreviations: LM, left main lesion; CTO, chronic total occlusion. | | | | | | | | |  |
|  |  |  |  |  |  |  |  |  |  |
|  |  |  |  |  |  |  |  |  |  |
